# µDrop: a system for high-throughput small-angle X-ray scattering measurements of microlitre samples

**DOI:** 10.1107/S1600576720014788

**Published:** 2021-02-01

**Authors:** Richard Haider, Barbara Sartori, Andrea Radeticchio, Marcell Wolf, Simone Dal Zilio, Benedetta Marmiroli, Heinz Amenitsch

**Affiliations:** a Institute of Inorganic Chemistry, Graz University of Technology, Stremayrgasse 9/IV, 8010 Graz, Austria; b Research Neutron Source Heinz Maier-Leibnitz (FRM II), Technical University of Munich, Lichtenbergstraße 1, 85748 Garching, Germany; c CNR-IOM – Istituto Officina dei Materiali, c/o Area Science Park1, Basovizza (TS), Italy

**Keywords:** small-angle X-ray scattering, sample changers, solution scattering, BioSAXS, microdrops

## Abstract

An automatic sample changer system for measurements of large numbers of liquid samples – the µDrop Sample Changer – is presented. It is based on a robotic arm equipped with a pipetting mechanism, which is combined with a novel drop-based sample holder. In this holder a drop of liquid is suspended between two parallel plates by surface tension. The absence of a transfer line benefits the cleaning, improving the background as well as making it faster and more efficient than most comparable capillary-based systems. The µDrop Sample Changer reaches cycle times below 35 s and can process up to 480 samples in a single run. Sample handling is very reliable, with a drop misplacement chance of about 0.2%. Very low sample volumes (<20 µl) are needed and repeatable measurements were performed down to 6 µl. Using measurements of bovine serum albumin and lysozyme, the performance of the instrument and quality of the gathered data of low and high concentrations of proteins are presented. The temperature of samples can also be controlled during storage and during measurement, which is demonstrated by observing a phase transition of a mesophase-forming lipid solution. The instrument has been developed for use in small-angle X-ray scattering experiments, which is a well established technique for measuring (macro-)molecules. It is commonly used in biological studies, where often large sets of rare samples have to be measured.

## Abstract 2

A new automatic sample changer based around a novel sample holder is presented for handling microlitre-sized sample drops.

## 1. Introduction   

Synchrotron beamlines are the preferred alternative when many samples are to be measured with X-ray techniques in a short time. With the vastly increased speed of measurements enabled by the high brilliance/flux, the time spent changing samples becomes the limiting factor. Additionally, the high brilliance of synchrotrons limits the maximum exposure duration due to radiation damage, which is often a problem with biological samples (Kuwamoto *et al.*, 2004 ▸).

Manual sample exchange is slow and tedious, generally requires more sample volume than strictly necessary for a measurement, and is prone to mistakes by the experimenter. Automation of this process is more efficient with respect to time and sample volume, and leads to more reliable and repeatable measurements. These criteria are essential in particular for small-angle X-ray scattering (SAXS) measurements of biological samples (BioSAXS). SAXS is a low-resolution structural method providing information on the dimension, supramolecular envelope and aggregation state (Kratky & Glatter, 1982 ▸). Therefore, in structural biology BioSAXS has become a frequently used technique, due to the development of new analysis methods, the use of noncrystalline samples and the versatility of sample conditions (Graewert & Svergun, 2013 ▸).

Automation of measurement procedures allows experimenters to spend their time more productively during beamtime sessions. It also enables mail-in experiments, where users can send their samples and perform the measurements via remote access. Removing the requirement for user groups to be physically present and thus the necessity of travel not only saves time and cost, but may be crucial in situations such as seen in the wake of the COVID-19 pandemic. Consequently, several beamlines have developed automatic sample changers for such applications (Table 1 ▸).

Here we present the µDrop Sample Changer, a new system based on a novel concept (Amenitsch *et al.*, 2015 ▸), which allows for improved performance in execution time, used volume and reliability (compare Table 1 ▸).

**Figure 1:**
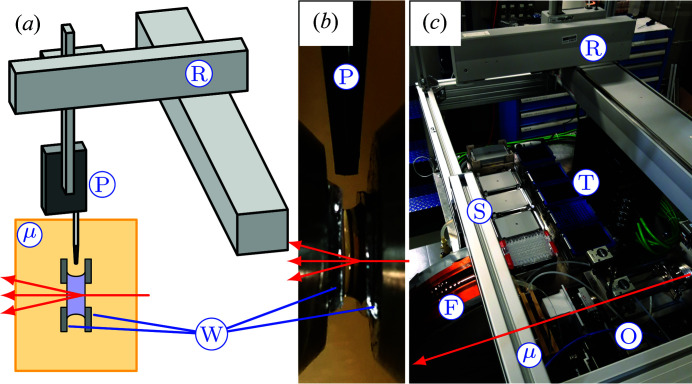
Working principle and overview of the µDrop Sample Changer. (*a*) Scheme of the instrument: the robotic arm (R) is equipped with a pipetting mechanism (P) that loads samples into the µDrop cell (µ). The drop is placed between two silicon plates (W) with X-ray-transparent windows, where it is held by surface tension. The X-ray beam (shown in red) passes through the windows, probing the sample. (*b*) Picture of a drop placed between the silicon nitride windows by pipetting from above (P). (*c*) Overview picture of the µDrop Sample Changer setup at the beamline. The X-ray path is shown by the red arrow. The robotic arm (R) takes samples from the sample trays (S) and moves them to the µDrop cell (µ). After the measurement the cell is automatically cleaned by dedicated hardware and the robot then places the next sample using a new tip taken from the tip trays (T).

The most common method used in other systems is to pump the sample into a capillary and use image recognition software for correct positioning. The µDrop Sample Changer works by pipetting a drop of sample between two parallel silicon wafers containing X-ray-transparent silicon nitride windows [Fig. 1 ▸(*a*)], where the drop is held by surface tension alone [Fig. 1 ▸(*b*)] and through which the sample is measured.

The µDrop system has several advantages over a capillary-based setup. As only a single drop is placed, the used volume is below 20 µl. In contrast, capillary setups often need a larger volume to work reliably (see Table 1 ▸), which could be problematic for very limited and/or expensive samples. In the µDrop Sample Changer, the sample is pulled into a disposable pipette tip and placed directly between the windows. A new tip is used for each measurement, preventing any cross-contamination that may occur along a tube-based sample transfer line. Additionally, the surface which has to be cleaned is a lot smaller than in capillary-based setups, making this step faster. Furthermore, the window distance can be adjusted to optimize the transmission of the sample (Kratky & Glatter, 1982 ▸), whereas capillary-based systems are limited to available capillary sizes.

In the following the µDrop Sample Changer and its operation, usage and performance are explained. The system was tested with a number of experiments: (i) to assess the repeatability, several successive cycles of water measurements were performed; (ii) dilution series of bovine serum albumin (BSA) and lysozyme were investigated to evaluate the quality of data and the performance from standard to low concentrations; (iii) handling of highly concentrated samples was tested by repeating partly the experiments reported by Zhang *et al.* (2012 ▸). There the scope was to study the influence of salts on the hydration shell and on the interaction of highly concentrated BSA in solution. (iv) For testing of thermal control, a temperature scan of a mesophase-forming lipid solution, namely 1,2-dipalmitoyl-*sn*-glycero-3-phosphocholine (DPPC), over thermally induced phase transitions (Koynova & Caffrey, 1998 ▸) was performed.

## 2. Setup description   

The µDrop Sample Changer was developed, optimized and tested at the Austrian SAXS beamline (Amenitsch *et al.*, 1998 ▸) at Elettra-Sincrotrone Trieste, Italy. The general concept and working principle of the system, however, are not dependent on the use of SAXS and the system can also be adapted to other experimental environments. At the beamline the flux density is of the order of 5 × 10^11^ photons s^−1^ mm^−2^; therefore, the sample holder is tailored for low flux density.

As the µDrop Sample Changer was designed to be compatible with the existing hardware, the beamline setup takes only about 1 h. This includes the alignment of the µDrop cell into the X-ray beam and the confirmation of the tip and sample tray positions, which have to be done when the instrument is newly set up.

Samples are placed in the instrument [Fig. 1 ▸(*c*)] in up to five standard 96-well plates (Carl Roth GmbH + Co. KG). The plate holders are thermalized by cooling/heating units (CPAC Ultraflat HT 2TEC, INHECO GmbH, Germany). One of them is additionally equipped with a shaker (Thermoshake, INHECO GmbH, Germany). The sample plates are sealed with plastic foils, to minimize evaporation and to avoid cross-contamination.

The samples are taken by a robotic arm (Cavro Omni Robot, Tecan Systems Inc., USA) equipped with a pipetting mechanism (Cavro Air Displacement Pipettor, Tecan Systems Inc., USA) which pulls them into a single-use tip (LiHa disposable tips, conductive, Tecan Systems Inc., USA). The instrument is supplied with 480 tips, matching the possible number of samples. With the current sampling rate it can run continuously for over 4 h, not including the measurement time.

The pipetting mechanism can automatically detect the liquid level within the well, which is particularly useful if available sample volumes are very low. The plastic foil covering the samples can interfere with the automatic detection, so a sample take-up from a specified height is also possible.

An automatic mixing mode is available, where sample is taken from one well and – instead of being measured – is dispensed into another well. This allows mixing of reactants on the fly, automatic creation of dilution series or storing of samples in a more stable condition, when in the measurement condition they would precipitate over time. The instrument can be set up to pump a defined volume in and out of the pipette tip several times to improve mixing, before continuing.

The robotic arm then takes a predefined volume of sample (5–20 µl) and moves it to the measurement cell. At concentrations above 100 mg ml^−1^ the drop handling begins to become unreliable, as the liquid uptake rate of the pipetting device is too fast. Thus, the possibility to slow down the take-up and placement of the sample has been added. Testing so far has revealed that the optimal pumping speed does not trivially correlate to the sample viscosity. The correct pipetting procedure for highly concentrated or particularly viscous samples is investigated for each sample class individually to ensure optimal drop placement.

In the µDrop cell the sample is dispensed between two rectangular windows [see Figs. 1 ▸(*a*) and 1 ▸(*b*)]. Each window consists of a 3 × 1 mm-large observation area made of 2 µm-thick silicon nitride prepared as reported by Bozzini *et al.* (2014 ▸), supported by a 1 mm-wide silicon frame. To enable changing of the window distance and thus sample thickness, the windows are glued onto stainless steel cylinders. These are mounted into the outer cell in such a way that they can slide and the distance be set using screws. Currently this cell is mounted in air, but provisions to connect the X-ray windows directly to the beamline vacuum path are planned. A detailed description of the outer cell is available in Appendix *A* [App appa].

The silicon frames are silanized (Pozzato *et al.*, 2006 ▸), increasing the hydrophobicity of the border with respect to the X-ray-transparent window to improve drop placement. Scattering angles of up to 20° can be reached with the presented setup, and a dedicated wide-angle cell is currently under development, which should reach angles of up to 70°.

After the measurement is completed, the detector image is processed to 1D data by *SAXSdog* (Burian *et al.*, 2020 ▸), the data reduction pipeline available at the Austrian SAXS beamline. This allows immediate analysis by investigating scattering patterns, integrated intensity, invariant, correlation length *etc*.

At the end of the exposures, the previous sample is flushed out with distilled water and funnelled through a tube to the liquid waste container, located below the cell. The windows are then cleaned with ethanol and rinsed with distilled water, which are discarded into the waste container. A total of 2 ml of ethanol and 10 ml of water is used as standard cleaning protocol. After cleaning, the windows are dried by two streams of nitrogen coming from different directions. During the cleaning and drying procedure the sample changer discards the used tip, mounts a new one and proceeds with the next sample. Depending on the position of the next tip and sample, the whole cycle takes up to 35 s.

The system supports the usage of up to three different cleaning solutions simultaneously. The common standard solution of Hellmanex III (Hellma Analytics, USA), if used extensively, was found to increase the hydrophilicity of the silicon nitride window, which is detrimental to the drop placement reliability. However, the standard cleaning procedure using 96% ethanol and distilled water was found to be effective for most samples. Additionally, the µDrop Sample Changer supports the use of two separate cleaning procedures, where one is set up for speed and the other can be optimized depending on the sample in question. Which of the two procedures is to be used can be selected for each sample individually. This allows for better background stability when measuring, for example, high concentrations without losing the speed of the standard cleaning procedure for other samples.

## 3. Program features   

The software to manage the autosampler is written in *LabView* (National Instruments, USA) in order to be compatible with the Austrian SAXS beamline control software. It allows users to load and run experiments defined *a priori*, but it is also possible to manually set up and execute single measurements as well as complex sequences. The experimental schedule can be adjusted easily and on the fly as the results of the first measurements become available.

**Figure 2:**
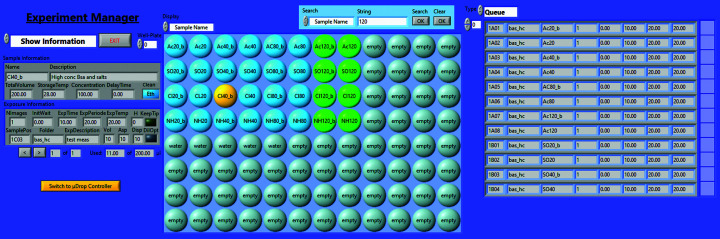
Screenshot of the *Experiment Manager* interface. The main feature is a representation of a well plate. It can display a variety of information, giving an intuitive overview of the samples being measured. The panel to the right shows a list of all pending measurements and the corresponding samples are also marked in the centre display (highlighted in blue). Detailed information on individual samples can be shown in the left panel by clicking the respective position (highlighted in yellow) in the central display.

The main user interface – the *Experiment Manager* – is shown in Fig. 2 ▸. In its centre a representation of a well plate displays detailed sample information for each cell of the well plate. Different plates can be cycled through and individual sample positions can be clicked on to provide the full set of associated information (left-hand side in Fig. 2 ▸). The details on the samples and the processing parameters can be loaded from an independent text file or entered manually, *e.g.* a new measurement can be added by double-clicking the respective sample position in the central array. A list of all pending measurements is also available (right-hand side in Fig. 2 ▸). The Sample Changer can execute programmed measurements even while the user is working with the *Experiment Manager*, and their progress is updated automatically.

The central display uses coloured highlighting to visualize the progress of the experiment (blue for planned measurements and red for the ongoing one). Coloration is also used to denote the currently selected sample (yellow), which is displayed on the left, and to enable searching and highlighting (green) samples based on specific parameters (*e.g.* a base name or a minimum remaining volume). A more detailed description of the program features is available in Appendix *B* [App appb]. The developed interface makes the operation of the µDrop Sample Changer simple and flexible, so that users can quickly use the instrument to its full potential.

## 4. Materials and methods   

### 4.1. SAXS measurements   

The experiments reported in the following were performed at the Austrian SAXS beamline (Amenitsch *et al.*, 1998 ▸) at Elettra-Sincrotrone Trieste, Italy. For the presented experiments, the beamline was operated at 8 keV and the X-ray beam was cut to a size of 2 × 0.4 mm (width × height). With the given beam size the flux is about 5 × 10^11^ photons s^−1^. The transmitted X-ray beam was measured using a photodiode mounted on the beamstop. Scattering images were recorded with a Pilatus3 1M detector (Dectris, Switzerland) at a sample-to-detector distance of 1.278 m. The 2D detector images were radially averaged, giving the scattering intensity as a function of the magnitude of the scattering vector *q*, *i.e.* a 1D scattering pattern *I*(*q*) (see Section 2), with 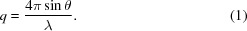
2θ is the scattering angle and λ the wavelength of the X-rays (0.154 nm). The setup allowed measurements of *q* values from 0.08 to 5.8 nm^−1^, calibrated using silver behenate (Huang *et al.*, 1993 ▸). The scattering patterns were normalized for fluctuations of the intensity of the primary beam and for transmission. Then the individual scattering patterns from all images of each sample were averaged and finally the respective backgrounds, treated in the same way, were subtracted. The resulting scattering patterns were converted to absolute intensity by rescaling the forward intensity, *I*(0), of a 4 mg ml^−1^ lysozyme measurement to the literature value (Mylonas & Svergun, 2007 ▸). The radius of gyration, *R*_g_, and the forward intensity, *I*(0), were extracted from the scattering patterns by performing a Guinier fit. The integrated intensity 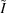, was calculated by summing the intensities over a defined *q* interval.

### 4.2. Sample preparation   

The experiments performed for the confirmation of the repeatability of measurements used ultra-pure water, Milli-Q grade (Merck KGaA, Darmstadt, Germany). For the minimum volume investigation BSA in HEPES (50 m*M*, pH 7.5) with a concentration of 11.5 mg ml^−1^ was used.

The samples for the concentration limit investigation were dilution series of BSA in HEPES (50 m*M*, pH 7.5) and of lysozyme in Tris–HCl (100 m*M*, pH 8), all from Sigma–Aldrich. The samples were prepared on site and their concentration was estimated using a UV–visible spectrophotometer (Cary60, Agilent Technologies, Santa Clara, CA, USA): 2, 1, 0.5, 0.3, 0.25, 0.17, 0.15 and 0.1 mg ml^−1^ for BSA, and 4, 2.4, 1, 0.5, 0.21 and 0.11 mg ml^−1^ for lysozyme. The samples and buffers were placed in a 96-well plate.

For the high-concentration test, BSA was prepared at 20, 40 and 80 mg ml^−1^, dissolved in solutions of different salts [1 *M* CH_3_COONa; 1 *M* NaC; 2 *M* (NH_4_)_2_SO_4_].

To demonstrate the possibility to thermally control the sample during measurements, 1,2-dipalmitoyl-*sn*-glycero-3-phosphocholine (DPPC; Larodan AB, SOLNA, Sweden) was prepared at 10 mg ml^−1^, suspended in Milli-Q water and dissolved via sonication (VCX130 Ultrasonic processor, Sonics & Materials Inc., Newton, USA) for 1 min at pulse amplitude 30% (2 s active, 3 s pause).

## 5. Results and discussion   

The measurements were conducted with a distance of 0.6 mm between the silicon nitride windows to achieve lower volumes. Except for the minimum volume tests, droplets of 11 µl were used for measurements, chosen to guarantee the complete filling of the observation cell and minimize the effects of evaporation. However, for water-based samples and commonly used exposure times (from a few seconds to 5 min) evaporation was found to be virtually negligible at room temperature (data not shown).

### 5.1. Repeatability and lowest volume   

The µDrop system works best with water-based samples, which includes the majority of biological samples. In this case, the optimal window distance for correct drop placement is 0.5–1 mm. In order to accommodate also other (in)organic solvent-based samples, the surfaces of the windows can be treated to be solventphilic. This aspect is still under investigation and thus not presented here.

**Figure 3:**
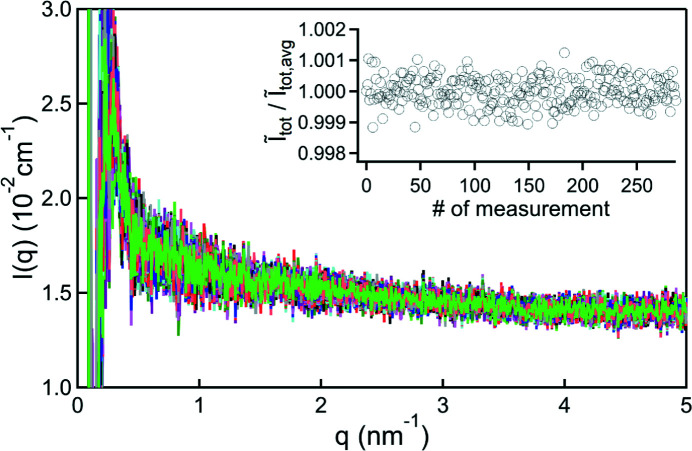
The background-subtracted scattering patterns of 288 individual drops of water. The inset shows the integrated intensities 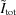 of each scattering pattern, determined over *q* = 0.5–5 nm^−1^ and normalized to the average 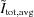. The variation among the values is dominated by the statistical noise, demonstrating the independence of the measurement from the drop placement and thus the excellent repeatability.

The results of the repeatability test are presented in Fig. 3 ▸, where the background-subtracted scattering patterns of 288 water droplets are shown. Each drop was measured with a single exposure of 20 s. In the inset the integrated intensity 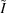
_tot_ (determined between *q* = 0.5 nm^−1^ and *q* = 5 nm^−1^) of each of the 288 scattering patterns is shown, normalized to the average 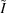
_tot,avg_. The data exhibit no drop placement dependent variation among the values. The fluctuations are rather dominated by the statistical noise, which can be reduced by longer exposure times or higher X-ray flux, making measurements very repeatable. These behaviours are not *q* dependent as integrating over sub-regions (*e.g.* only high *q*) gives the same results (not shown).

An investigation conducted by successively placing 40 droplets of 11.5 mg ml^−1^ BSA of progressively smaller volumes (10, 9, 8, 7, 6 and 5 µl, each measured three times for 20 s) showed no volume dependence of the rate of successful drop placement. The extent of the deviations of the individual scattering patterns from the average increases well over 5% at 5 µl (data not shown).

### 5.2. Dilution series   

To investigate the lower concentration limit several drops were placed for each concentration (eight for BSA, 12 for lysozyme), coming from different wells. A buffer measurement was performed preceding each sample measurement. In every measurement ten detector images, 20 s each, were taken. Guiner fits were performed as straightforward classification of the measurement quality. In the analysis of lysozyme, an increase of *I*(0) greater than the statistical fluctuation was observed from the sixth exposure on, indicating radiation damage, so only the first five were used. This identification was done manually, but an automated procedure is under investigation, following the approach described by Schroer *et al.* (2018 ▸) and Franke *et al.* (2015 ▸).

Incorrectly placed drops can easily be identified from their scattering patterns. Among the 272 measurements performed for the dilution series, three drops were not placed properly: one sample from the 0.3 mg ml^−1^ BSA set, which was discarded, and two buffers. For those two, the buffer measurement after the respective sample was used for background subtraction.

**Figure 4:**
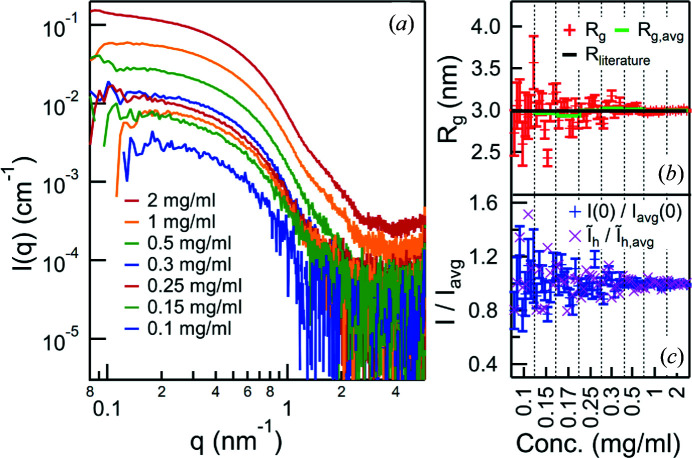
Dilution series of BSA. (*a*) Averaged scattering patterns of different concentrations, vertically shifted for clarity. (*b*) *R*_g_ and (*c*) *I*(0) (normalized by the average of each concentration) as extracted from the individual measurements via Guinier fit. Due to the aggregation of the BSA, Guinier fitting could not be done in the recommended region of 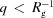. The shown values come from fitting a region (*q* = 0.48–0.70 nm^−1^) that is linear in the Guinier plot. While the individual *R*_g_ values show a strong fluctuation at low concentrations, the averages for each concentration, shown in green, are very consistent. The literature value (Mylonas & Svergun, 2007 ▸) is given by the black line. In (*c*) also 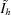, the scattering intensity integrated over *q* = 3–5 nm^−1^, is shown (normalized by the average of each concentration), highlighting the stability also in the high-*q* range.

Figs. 4 ▸(*a*) and 5 ▸(*a*), respectively, show the average of all collected scattering patterns of the BSA and lysozyme of each concentration. Even though the lowest concentrations suffer from a high noise level individually, the averaged patterns are still in line with the other results. The BSA data show some signs of aggregation at lower *q*, which is attributed to the long storage time at room temperature.

The values found for *R*_g_ and *I*(0) are shown in Figs. 4 ▸(*b*), 4 ▸(*c*), 5 ▸(*b*) and 5 ▸(*c*) for all the measured drops. The *I*(0) values are normalized to the average of each concentration, to highlight the fluctuation between individual measurements. Due to the aggregation of the BSA the fitting was done in a linear region in the Guinier plot above the recommended limit of 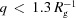 (shown in Fig. 4 ▸ are the results from fitting *q* = 0.48–0.70 nm^−1^). Even though the radius of gyration evaluation was performed out of the area of validity, its values are very sensitive to the alteration of the scattering pattern during the experiments. Quantitative results are given for the lysozyme measurements, where the Guinier regime extended up to *q* = 2 nm^−1^. The average values of each concentration are listed in Table 2 ▸. The resulting *R*_g_ deviates from the 1.43 ± 0.04 nm reported in the literature (Mylonas & Svergun, 2007 ▸), but it is consistent over all measurements. This deviation was attributed to the different buffer composition (Hirai *et al.*, 2012 ▸) and to a slight aggregation that was revealed by a calculation of the pair distribution function (data not shown). To give an indicator of the stability at high values of *q* the scattering intensity 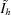, integrated from *q* = 3 nm^−1^ to *q* = 5 nm^−1^ and normalized by the average of each concentration, is also shown in Figs. 4 ▸(*c*) and 5 ▸(*c*).

The 272 measurements for the BSA and lysozyme dilution series presented here took just 14 h, of which 11 h were pure measurement time. Cleaning the cell and placing the next sample made up only about 20% of the total time and subsequent improvements of the system have reduced this time even further. Approximately 99% of the sample drops were placed such that usable data could be measured. On the basis of findings during these experiments the sample changer system has been improved and more recent investigations show a misplacement chance of only about 0.2% (data not shown).

### 5.3. High concentrations   

**Figure 6:**
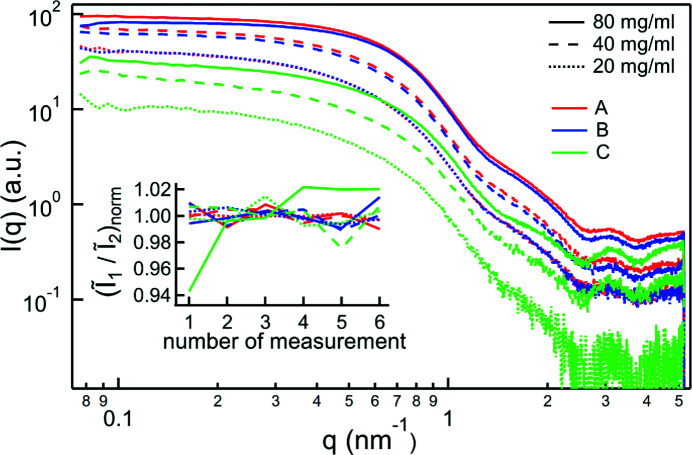
Scattering patterns of 20, 40 and 80 mg ml^−1^ BSA in salt solutions: A: 1 *M* CH_3_COONa; B: 1 *M* NaCl; C: 2 *M* (NH_4_)_2_SO_4_. The inset shows the ratio between two integrated intensities 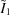 and 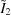, integrated over *q*_1_ = 0.5–0.8 nm^−1^ and *q*_2_ = 0.8–1.1 nm^−1^, respectively, each normalized by the average for that combination. This ratio highlights changes in the shape of the scattering patterns and thus serves as a simple indicator for the stability of the measurements over time.

For the test at high concentrations, BSA in combination with different salts was measured, changing first the salt solution and then increasing the protein concentration. The entire measurement set was repeated six times, exposing each sample for 10 s. The averaged results of all six measurements for each combination are shown in Fig. 6 ▸. With a total exposure time of 1 min per combination, high-quality scattering patterns could be achieved.

The inset of Fig. 6 ▸ shows for each of the six individual measurements of each salt and concentration combination the ratio of two integrated intensities 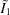 and 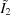, integrated over *q*_1_ = 0.5–0.8 nm^−1^ and *q*_2_ = 0.8–1.1 nm^−1^, respectively, each normalized by the average for that combination. This ratio serves as a simple indicator of the stability of the gathered data over time, as a change in this ratio implies a change in the shape of the scattering pattern. Most of the measurement series exhibit only statistical fluctuation, with only the highest concentrated BSA in the (NH_4_)_2_SO_4_ solution showing an increase over time. This instability is believed to be due to the effect of SO_4_^2−^ in combination with the high concentration of the protein, which leads to the formation of aggregates over the course of the experiment (Zhang *et al.*, 2012 ▸).

### 5.4. Thermal control   

The temperature of the µDrop cell can be set independently from the well plates. Placed samples equilibrate quickly (<20 s) to the temperature of the cell. A sample could thus, for example, be stored at low temperature and then measured at a biologically relevant one.

**Figure 7:**
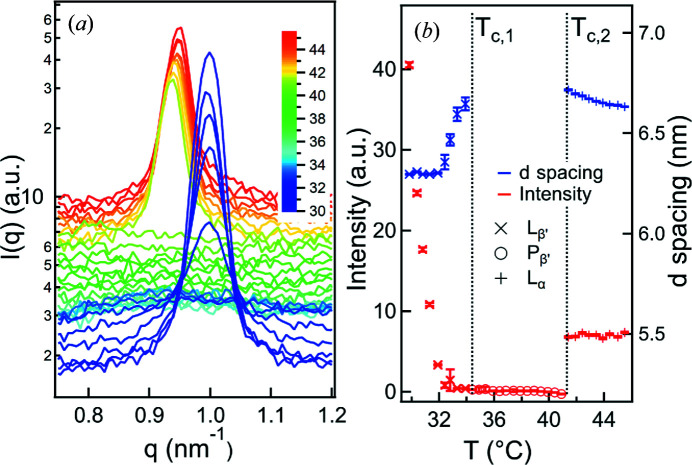
Temperature scan of DPPC. (*a*) Scattering pattern obtained at different temperatures showing the phase transition 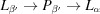 (shown is the first-order diffraction peak, shifted vertically for clarity). (*b*) The intensity and *d* spacing of the individual peaks for each phase versus temperature. The *d* spacing of the 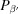 phase is omitted as it cannot be determined reliably. The clear and sharp phase transition 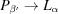, observed at the expected 41.3°C, highlights the precise thermal control possible with the instrument.

It is also possible to change the temperature during the measurement. To demonstrate this, a drop of DPPC was placed in the µDrop cell at 30°C. The cell was then gradually heated at a rate of 0.5°C min^−1^ and repeated measurements were taken (one 5 s exposure every minute). The DPPC undergoes a transition from the 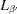 gel bilayer phase first to the 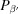 ripple phase and then to the *L*_α_ liquid crystalline phase (Pabst *et al.*, 2004 ▸), which occur at temperatures *T*_c,1_ = 34.4°C and *T*_c,2_ = 41.3°C, respectively (Koynova & Caffrey, 1998 ▸). Fig. 7 ▸(*a*) shows the scattering patterns at different temperatures, while Fig. 7 ▸(*b*) gives the intensity and *d* spacing of the first-order peak shown in Fig. 7 ▸(*a*), obtained via a Gaussian fit, highlighting the phase transitions. The first transition temperature *T*_c,1_ varies with the sample history and preparation, and is within an acceptable range; in contrast, the second transition temperature *T*_c, 2_ is clearly identifiable between 40.9 and 41.4°C. This demonstrates the precise thermal control of the instrument.

## 6. Conclusion   

A new instrument – the µDrop Sample Changer – was developed, combining a novel µl-drop-based sample holder with a robotic arm to create a sample changer capable of automatically measuring minute volumes. The device can reliably handle liquid solutions in the range of 5–20 µl. A cleaning and loading cycle (not including the measurement time) takes less than 35 s. The instrument can measure up to 480 samples in a row. The µDrop Sample Changer is thus a great aid for experiments where large numbers of samples have to be measured, such as protein investigations. The system is very reliable with water-based samples (∼0.2% misplacement chance), and by treating the windows to be solventphilic a broader range of (in)organic solvents may be accessed.

Using exemplary measurements of BSA and lysozyme, it was demonstrated that high-quality SAXS data can be obtained. Measurements not only are highly repeatable but also give good results even at low concentrations. The instrument can handle samples with concentrations higher than necessary for most protein investigations.

The custom software written to control the sample changer makes executing large-scale measurement series very simple. It is also designed for individual investigations and for the manual creation of short exploratory series. This allows quick and easy preliminary measurements to optimize the exposure parameters. The entire experimental schedule can then be adjusted, should new or unexpected findings be made.

The automatic µDrop Sample Changer presented here is already available for users at the Austrian SAXS beamline at Elettra-Sincrotrone Trieste, Italy.

## Figures and Tables

**Figure 5:**
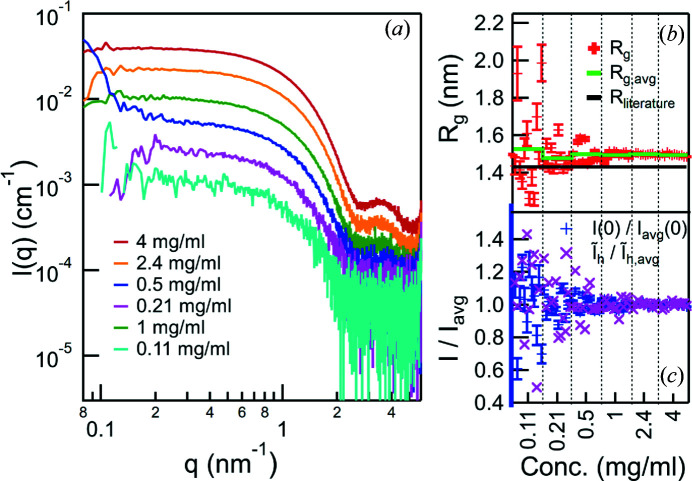
Dilution series of lysozyme. (*a*) Averaged scattering patterns of different concentrations, vertically shifted for clarity. (*b*) *R*_g_ and (*c*) *I*(0) (normalized by the average of each concentration) as extracted from the individual measurements via Guinier fit. While the individual *R*_g_ values show a strong fluctuation at low concentrations, the averages of the different concentrations, shown in green, are in good agreement with each other. The offset from the literature value (Mylonas & Svergun, 2007 ▸) given by the black line is attributed to the different buffer composition. In (*c*) also 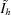, the scattering intensity integrated over *q* = 3–5 nm^−1^, is shown (normalized by the average of each concentration), highlighting the stability also in the high-*q* range.

**Figure 8:**
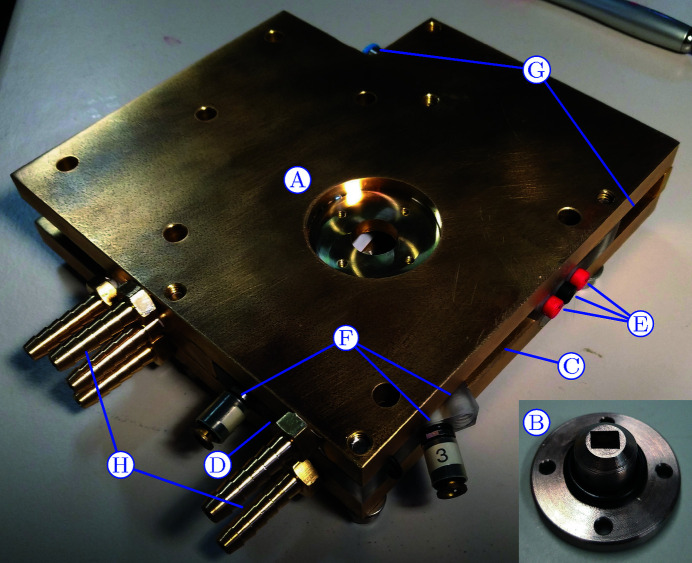
The outer cell of the µDrop Sample Changer. (A) The hole containing (B) the window holding cylinder. (C) The opening for the sample placement. (D) Housing for a camera. (E) Connectors for the internal lighting. (F) Connectors for the cleaning liquids and drying nitrogen. (G) The outlets for the waste liquid and air. (H) Connectors for the thermal control fluid.

**Figure 9:**
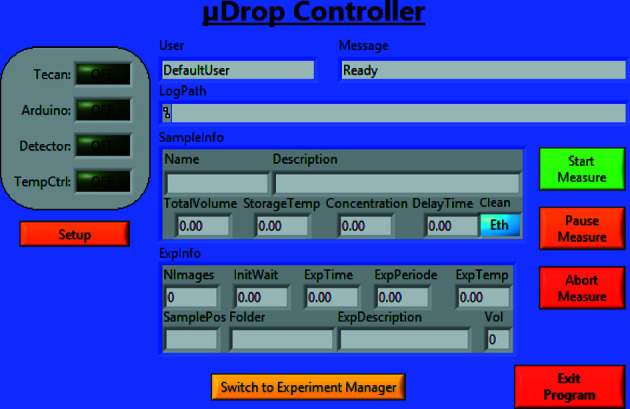
Screenshot of the *µDrop Controller*. This interface is used to start and stop the sample changer. While a non-empty ‘Queue’ exists in the *Experiment Manager*, this program keeps the instrument operating. The state of the instrument and information on the currently handled sample are displayed. Further buttons give access to sub-programs, switch to the *Experiment Manager*, abort the operation in case of emergency or stop the program itself.

**Table 1:** Comparison of the presented µDrop and different automatic sample changer systems in operation at other beamlines Beamlines used in comparison: the Australian Synchrotron SAXS/WAXS beamline (Ryan *et al.*, 2018 ▸), the SWING beamline of Synchrotron SOLEIL (David & Pérez, 2009 ▸), beamline 4-2 of the Stanford Synchrotron Radiation Lightsource (SSRL) (Martel *et al.*, 2012 ▸), SIBYLS beamline (12.3.1) of the Advanced Light Source (ASL) at Lawrence Berkeley National Laboratory (Classen *et al.*, 2013 ▸), the BM29 beamline at the European Synchrotron Radiation Facility (ESRF), the P12 beamline at the PETRA-III synchrotron (EMBL@PETRA-III) and the I22/B21 beamlines at Diamond Light Source (Round *et al.*, 2015 ▸). The values are taken from the referenced articles and the web pages of the respective groups.

Autosampler beamline	Volume	Time	Sample holder
Australian SAXS/WAXS	100 µl	420 s	Quartz capillary
SWING at SOLEIL	10–50 µl	240 s	Glass capillary
Beamline 4-2 at SSRL	20–30 µl	180 s	Quartz capillary
SIBYLS at ALS	24–30 µl	140 s	Pipetted to wells
BM29 at ESRF, P12 at PETRA-III, I22/B21 at Diamond	5–250 µl	50 s	Quartz capillary
Austrian SAXS at Elettra	5–20 µl	35 s	SiNx windows

**Table 2:** *R*_g_ and *I*(0) of the lysozyme measurements, extracted by Guinier fit and averaged for each concentration

Concentration (mg ml^−1^)	*R*_*g*_ (nm)	*I*(0) (10^−2^ cm^−1^)
4.00 ± 0.05	1.494 ± 0.003	4.093 ± 0.010
2.40 ± 0.05	1.498 ± 0.003	2.323 ± 0.008
1.00 ± 0.05	1.494 ± 0.007	1.062 ± 0.009
0.50 ± 0.05	1.499 ± 0.017	0.571 ± 0.008
0.21 ± 0.05	1.477 ± 0.028	0.260 ± 0.009
0.11 ± 0.05	1.527 ± 0.088	0.117 ± 0.008

**Table 3:** A list of the parameters of the sample changer software, some of which are optional The first set is the sample information; the second set consists of the measurement variables. The required parameters are marked with an asterisk (*); the others can also be filled with dummy values.

Parameter	Range	Description
Position	1 A01–5 H12	Part of file mainly for readability
Name*	1–10 ASCII	Base name of the created files
Description	–	Additional sample information
TotalVolume*	<300 µl	Provided sample volume
StorageTemp	4–40°C	Well-plate temperature
Concentration	0–100	For easier manual creation of runs
Delay	≥0 s	Sample-based exposure delay
		
Cleaning	0 or 1	Standard or extended cleaning
SamplePos*	1 A01–5 H12	Sample to be measured
Folder*	1–31 ASCII	For organization of sub-groups
NImages*	1–65535	Number of images per sample
InitWait	≥0 s	Measurement-based exposure delay
ExpTime*	≥1.05 ms	Exposure time per image
ExpPeriod*	≥2 ms	Time from one image to next
ExpTemp	4–60°C	Measurement temperature
Description	–	Additional measurement information
Volume*	1–256 µl	Volume to be measured
AspVol	1–256 µl	Step size of sample take-up
DispVol	1–256 µl	Step size of sample placement
Height	≥0 µm	Drop placement height offset
KeepTip	0 or 1	Reuse pipette tip
DilOpt	0 or 1	Use automatic dilution option

## Appendix A The µDrop cell

The core of the µDrop Sample Changer is the eponymous µl-drop-based sample holder. It is contained in an outer cell built from brass, shown in Fig. 8 ▸. There are a number of features marked: at its centre there is a hole (A) in which a metal cylinder (B) can be placed that carries the silicon frame with the silicon nitride window. A similar hole for a second cylinder and window is present on the opposite side. The droplet is placed there by a robotic arm with a pipetting mechanism, which enters the cell through the opening (C) at the top of the cell, and held in place by surface tension. The drop positioning can be observed via an endoscopic camera (BS-19+ USB endoscope, Voltcraft, Switzerland) placed in the opening (D). LEDs (3.7 V white LED, Nichia, Japan) inside the cell (E) improve visibility. After the measurement, the observation windows are cleaned by different fluids and subsequently dried by two separate nitrogen streams. The connectors (F) for these are visible at the side and top of the cell. The liquid waste and excess nitrogen can exit the cell at the bottom and top of the cell (G), respectively. The cell can be connected (H) to a thermostated water bath for thermalization of the sample.

## Appendix B Software details

The *Experiment Manager* (Fig. 2 ▸), the main interface to operate the system, has already been described in the main text. Here a more detailed discussion of its features and their usage is presented, and the program to operate the hardware – the *µDrop Controller* – introduced.

The *µDrop Controller* (Fig. 9 ▸) is used to start, pause and, in case of an emergency, abort the operation of the µDrop Sample Changer. While running it always shows the entirety of available information on the current sample and measurement, as well as the progress of the operation. The program also displays the state of the various instruments, *i.e.* the robotic arm, the microcontroller (Arduino Uno Rev3, Arduino, USA, plus Relay Shield v3.0, Seeed Studio, China), handling cleaning and electronics, the detector(s), and the optional temperature controller. These can all be initialized and configured from the respective sub-programs available by pressing the ‘Setup’ button. For some instruments also low-level interfaces are available, mainly useful for troubleshooting and occasional non-standard operations. Additionally, assistant programs can be accessed to adjust the cleaning protocol if needed or to configure the tip and sample plate positions. This interface is mainly used during setup and to start the Sample Changer.

The user will work mainly with the *Experiment Manager* (Fig. 2 ▸). If the lists of samples and corresponding exposures have been completely prepared beforehand and are used without modification, the information can simply be loaded in and the experiment started within a few seconds. Most users, however, opt to do several preliminary test measurements and then design a custom measurement run based on their findings. In this way they can take full advantage of the functionalities that the *Experiment Manager* provides.

To perform an experiment the program needs data on the loaded samples and on the planned measurements. The sample information consists of parameters that will normally not change, like the names and their order in the well plates, the volumes provided, the concentrations *etc.* The measurement variables define, for example, the order of the samples, the exposure times, the temperatures *etc.* These can often change according to results of previous measurements. A complete list of all parameters is given in Table 3 ▸.

The information necessary for an experiment can be provided to the program by loading the required parameters from text files, which can be produced in any spreadsheet program. This is particularly useful in the case of the sample information, which is best written alongside the filling of the well plates and can be expected to stay the same during the experiment. Alternatively the parameters can be entered manually, which is especially useful for initial investigations, where the exposure parameters can be expected to change on the basis of preliminary findings. For this, the program is designed to facilitate the manual creation of measurement sequences in an easy and flexible manner.

New exposures can be added simply by double-clicking the position of the desired sample in the well-plate representation in the centre of the *Experiment Manager* interface. This display shows the information on all samples in one well plate. If more well plates are in use, they can be cycled through. The type of information, such as sample name, remaining volume, position in the measurement sequence *etc.*, can be selected above the display. A single click on a well-plate position (which is then highlighted in yellow) displays on the left side of the interface the information on the corresponding sample, as well as the parameters of all different measurements of this sample.

The *Experiment Manager* has three modes: ‘Show Information’ mode (the one seen in Fig. 2 ▸), which serves to prevent unintentional modification of provided data, ‘Manage Samples’ mode, in which the information on the highlighted sample can be modified, new sample entries created, and the sample information saved and loaded, and ‘Create Sequence’ mode, in which measurement variables can be entered and new measurements queued up.

As the µDrop Sample Changer can continue operating while the user is working to create new experimental runs, the new sample measurements are not immediately added to the list of pending exposures, called the ‘Queue’. They are instead first collected in the ‘Selected’ list. Once a sequence has been created, it can be moved to the ‘Queue’ with the press of a button.

The µDrop Sample Changer continuously works through the ‘Queue’ until it is empty. The currently measured sample is highlighted in red. Once the measurement is complete, the ‘Queue’ entry is moved to the ‘Done’ list, which can also be displayed, showing all measurements that have already been performed. The entirety of parameters for each measurement are also collected and logged automatically, making the whole process easily reproducible.

To further ease the manual creation of experiments, a search function is provided as well. For example, one can search for all samples of a given base name (*e.g.* all starting with ‘bsa’), with a specific concentration, measured above a certain temperature, with a minimum volume remaining *etc.* The positions of all samples fitting the specified criterion are then highlighted in green.
